# Bacterial and viral pathogen spectra of acute respiratory infections in under-5 children in hospital settings in Dhaka city

**DOI:** 10.1371/journal.pone.0174488

**Published:** 2017-03-27

**Authors:** Golam Sarower Bhuyan, Mohammad Amir Hossain, Suprovath Kumar Sarker, Asifuzzaman Rahat, Md Tarikul Islam, Tanjina Noor Haque, Noorjahan Begum, Syeda Kashfi Qadri, A. K. M. Muraduzzaman, Nafisa Nawal Islam, Mohammad Sazzadul Islam, Nusrat Sultana, Manjur Hossain Khan Jony, Farhana Khanam, Golam Mowla, Abdul Matin, Firoza Begum, Tahmina Shirin, Dilruba Ahmed, Narayan Saha, Firdausi Qadri, Kaiissar Mannoor

**Affiliations:** 1 Infectious diseases Laboratory, Institute for Developing Science and Health Initiatives, Mohakhali, Dhaka, Bangladesh; 2 Genetics and Genomics Laboratory, Institute for Developing Science and Health Initiatives, Mohakhali, Dhaka, Bangladesh; 3 Department of Paediatric Medicine, KK Women’s and Children’s Hospital, Singapore, Singapore; 4 Department of Virology, Institute of Epidemiology, Disease Control and Research, Mohakhali, Dhaka, Bangladesh; 5 Department of Virology, Mymensingh Medical College, Dhaka, Bangladesh; 6 Department of Enteric and Respiratory Infectious Diseases, Infectious Diseases Division, International Centre for Diarrhoeal Disease Research, Bangladesh, Mohakhali, Dhaka, Bangladesh; 7 Department of Pediatrics, Suhrawardy Medical College Hospital, Dhaka, Bangladesh; 8 Department of Child Health, Dhaka Medical College Hospital, Dhaka, Bangladesh; 9 Department of obstetrics and gynecology, Bangabandhu Sheikh Mujib Medical University, Dhaka, Bangladesh; 10 Pediatric Neurology, National Institute of Neurosciences & Hospital, Dhaka, Bangladesh; Kliniken der Stadt Köln gGmbH, GERMANY

## Abstract

The study aimed to examine for the first time the spectra of viral and bacterial pathogens along with the antibiotic susceptibility of the isolated bacteria in under-5 children with acute respiratory infections (ARIs) in hospital settings of Dhaka, Bangladesh. Nasal swabs were collected from 200 under-five children hospitalized with clinical signs of ARIs. Nasal swabs from 30 asymptomatic children were also collected. Screening of viral pathogens targeted ten respiratory viruses using RT-qPCR. Bacterial pathogens were identified by bacteriological culture methods and antimicrobial susceptibility of the isolates was determined following CLSI guidelines. About 82.5% (n = 165) of specimens were positive for pathogens. Of 165 infected cases, 3% (n = 6) had only single bacterial pathogens, whereas 43.5% (n = 87) cases had only single viral pathogens. The remaining 36% (n = 72) cases had coinfections. In symptomatic cases, human rhinovirus was detected as the predominant virus (31.5%), followed by RSV (31%), HMPV (13%), HBoV (11%), HPIV-3 (10.5%), and adenovirus (7%). *Streptococcus pneumoniae* was the most frequently isolated bacterial pathogen (9%), whereas *Klebsiella pneumaniae*, *Streptococcus* spp., *Enterobacter agglomerans*, and *Haemophilus influenzae* were 5.5%, 5%, 2%, and 1.5%, respectively. Of 15 multidrug-resistant bacteria, a *Klebsiella pneumoniae* isolate and an *Enterobacter agglomerans* isolate exhibited resistance against more than 10 different antibiotics. Both ARI incidence and predominant pathogen detection rates were higher during post-monsoon and winter, peaking in September. Pathogen detection rates and coinfection incidence in less than 1-year group were significantly higher (P = 0.0034 and 0.049, respectively) than in 1–5 years age group. Pathogen detection rate (43%) in asymptomatic cases was significantly lower compared to symptomatic group (P<0.0001). Human rhinovirus, HPIV-3, adenovirus, *Streptococcus pneumonia*, and *Klebsiella pneumaniae* had significant involvement in coinfections with P values of 0.0001, 0.009 and 0.0001, 0.0001 and 0.001 respectively. Further investigations are required to better understand the clinical roles of the isolated pathogens and their seasonality.

## Introduction

Acute respiratory infections (ARIs), in particular, pneumonia, remain the persistent and pervasive deterrent to public health. It is responsible for substantial morbidity and mortality worldwide, especially in children <5 years of age. But the death rate due to ARI is mostly concentrated in developing regions like Africa and Southeast Asia. The World Health Organization (WHO) estimates that acute respiratory infections account for 1.9 to 2.2 million childhood deaths annually, with 70% occurring in Africa and Southeast Asia [1, 2]. The definition of ARI includes all infections of the respiratory tract. However, a majority of respiratory deaths are attributed to acute lower respiratory infections (ALRIs). The incidence of ALRIs in children aged less than 5 years is estimated to be 0.22 episodes per child-year, with most cases occurring in India, China, Pakistan, Bangladesh, Indonesia and Nigeria [3, 4].

The etiological agents for ARIs include bacteria, virus and fungi. The most commonly associated ARI-causing bacterial organisms are *Streptococcus pneumoniae*, *Haemophilus influenzae*, *Staphylococcus aureus* etc. Other atypical bacteria such as *Mycoplasma pneumoniae*, *Chlamydophila pneumoniae*, and *Klebsiella pneumaniae* are less frequently reported [5, 6]. Respiratory syncytial virus (RSV), influenza viruses type A and B, human parainfluenza viruses type 1, 2, and 3 (HPIV-1, HPIV- 2 and HPIV-3), adenoviruses (ADV) and human metapneumoviruses (HMPV) are most frequently detected in children with ARIs [7, 8]. Respiratory viruses are considered as major contributors to ARI in children and they are associated with almost 60% of ALRIs [9].

Although numerous pathogens are associated with ARIs, the clinical manifestations of ARIs are almost similar in all cases, irrespective of causative agents. So, detection of the potential causative agents is the prerequisite for proper treatment and viral detection can reduce the irrational and overuse of antibiotics. ARIs due to bacterial infections have become a global concern especially because of the emergence of an increasing number of multi-drug resistant bacteria. Although some studies have examined the viral etiology of ARIs in the under-five children in Bangladesh, information is lacking on bacterial pathogens and their antibiotic resistance/sensitivity pattern [10, 11]. Lack of information regarding etiological agents influences the use of inappropriate and broad spectrum antibiotics which in turn fosters antimicrobial resistance. The purpose of this study was to examine both viral and bacterial pathogens of ARIs among under-five children of Bangladesh in hospital settings over a period of 1 year. The viral pathogens were identified using RT-qPCR targeting 10 common respiratory viruses and microbiological culture method was used to examine pathogenic bacterial spectrum and determine their antibiotic susceptibility pattern.

## Methods and materials

### Study population

This is a prospective investigation of acute respiratory infections in under-five children in hospital-care settings in Dhaka city. A total of 200 patients who had acute respiratory infections (ARIs), as manifested by clinical signs and symptoms and/or chest X-ray report suggestive of pneumonia admitted to the pediatric units of Dhaka Medical College Hospital and Shaheed Suhrawardy Medical College Hospital, were enrolled in this study over a period of 1 year from August 2014 to July 2015. 30 (15 from each of 2 hospitals) asymptomatic under-five children who were admitted to the hospital because of complications other than respiratory infections were also enrolled during the study period. Prior to enrollment, a written informed consent was obtained from each child’s parents or legal guardians. Patients with a history of chronic respiratory infections or patients whose parents’/guardians refused to give consent were excluded from the study. In addition to name, address, height, weight and temperature, patients’ demographic data such as age and gender were recorded on a standard form at the time of specimen collection. Vital signs including heart rate and respiratory rate were also recorded at the same time. Nasal swab specimens were collected from each participant. The study was conducted in the Institute for Developing Science and Health Initiatives (IDESHI), Dhaka, Bangladesh. This study was ethically approved by the ethics review committee of Bangladesh Medical Research Council (BMRC).

### Specimen collection

Nasal swabs (NSs) were collected from enrolled participants by trained medical assistants. A sterile cotton flocked swab was soaked in saline and then placed approximately 1 to 1.5 cm into the nostrils and rotated against the anterior nasal mucosa for 3 seconds. The procedure was repeated using another swab. One of the 2 NSs was placed into viral transport media (VTM) (DMEM, Gibco-BRL, Life Technologies, Paisley, Scotland; penicillin 10,000 U/ml, and streptomycin 10,000 IU/ml, BioWhittaker, MA; NaHCO_3_; HEPES buffer, 1M, Gibco; L-Glutamate 200 mM; Fungizone (Amphotericin B) 250 μg/ml; bovine serum albumin, Fraction V, 7.5%, Gibco) for detection of viral pathogens, whereas the other one was placed in STGG (skim milk tryptone glucose glycerol) media for bacterial culture. The collected specimens were immediately placed at 2–8°C and transported to IDESHI laboratory. The specimens had been collected before the patients received any antibiotic treatment at the hospital. Specimens that had been collected in STGG media were subjected to a bacterial culture within 4 hours of collection, whereas specimens in VTM were stored at -70°C for later detection of viral pathogens by RT-qPCR.

### Detection of bacterial pathogens

The nasal swabs in STGG media were enriched for 4 hours in Todd Hewitt media supplemented with 20% rabbit serum. The bacterial pathogens were then isolated using selective media including MacConkey Agar, Blood Agar with Gentamicin, and Chocolate Agar. After morphology-dependent selection, colonies were identified by specific tests where necessary such as, optochin sensitivity and bile solubility tests for *Streptococcus pneumoniae*, tests for requirement of growth factors X, V and XV for *Haemophilus influenzae* and appropriate biochemical tests including TSI (Triple Sugar Iron), Citrate, MIU (Motility Indole Urea) media, and Analytical Profiling Index API^®^ 20E (bioMérieux, St. Louis, USA) for Enterobacteriaceae like *Klebsiella pneumoniae*. Profiling of antimicrobial susceptibility/resistance was performed by the modified disc diffusion method and the bacterial strains were identified as either sensitive or resistant to an antibiotic based on the diameter of inhibition zone interpretative chart, published in Clinical and Laboratory Standard Institute (CLSI) guidelines 2014.

### Virus pathogen identification

Viral nucleic acids were extracted from nasal swab specimens that had been collected in VTM using PureLink^®^ Viral RNA/DNA Mini Kit (Invitrogen, CA, USA). The extracted nucleic acids were then subjected to single-plex reverse transcription qPCR (RT-qPCR) for detection of respiratory viruses including human rhinovirus (HRV), respiratory syncytial virus (RSV), influenza A and B, human parainfluenza viruses type 1 to 3 (HPIV1, HPIV2, HPIV3), human metapneumovirus (HMPV), adenovirus, and human bocavirus (HBoV). The RT-qPCR was done using AgPath-ID^™^ One-Step RT-PCR Kit (Invitrogen, CA, USA) and CFX96 Touch^™^ Real-Time PCR detection system (Bio-Rad, CA, USA). The results of RT-qPCR were considered positive if an exponential fluorescence curve could cross the assigned threshold at C_t_<40.0. Previously published primers and probes were used for viral pathogen detection by RT-qPCR [12–15].

### Statistical analysis

Two-tailed chi-square test (two by two table) was performed using http://www.openepi.com/ website to calculate the prevalence of the identified pathogens and coinfection frequencies in different age groups. The relative risk ratio (RR) and odds ratio (OR) were estimated at 95% confidence interval (CI). Fisher's exact test using GraphPad Prism 5.0 was done for comparison of pathogen frequencies between symptomatic and asymptomatic children as some of the values were less than five. A p-value of less than 0.05 was considered significant.

## Results

### Demographic characteristics of study population

Prior to enrollment, all the children were examined for clinical signs and symptoms by attending physicians to suspect ARIs. The suspected patients were then subjected to chest X-ray. 62% (n = 124) of hospitalized symptomatic children were male, while the rest 38% were female. The mean age of the symptomatic children was 9.6 months and 47% (n = 94) of children were under six months of age and 78.5% (n = 157) were less than one year old. On the other hand, the mean age of the asymptomatic children was 12.62 months. All the children including their parents/guardians were residents of Dhaka city.

### Rate of ARI patient enrollment and specimen collection during the study period

**Fig 1:**
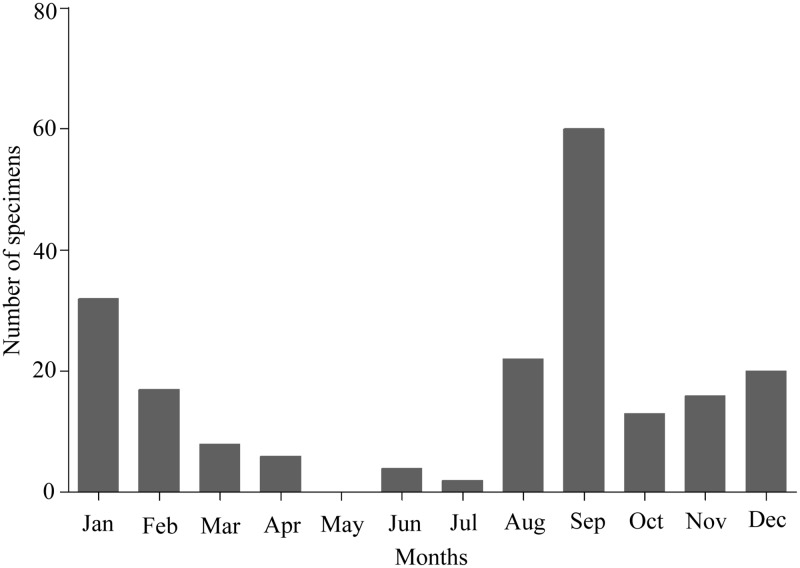
The frequency of specimen collection during the study period. Y-axis indicates the number of specimens, whereas X-axis indicates months of the year.

A total of 200 children with ARI illness were enrolled over 1 year period from August 2014 to July 2015. Fig 1 demonstrates the month-wise rate of specimen collection from enrolled patients. The rate of specimen collection reached a peak in September (30%; 60/200)), followed by a sharp decline in October (6.5%; n = 13). After October, a gradual increase in the rate of specimen collection was observed during the months of November, December, and January, with the rate of 8% (n = 16), 10% (n = 20), and 16% (n = 32), respectively.

Again, after January, there was a gradual decline in specimen collection rate from February (8.5%; n = 17) to May (0%), followed by 2% (n = 4) and 1% (n = 2) collection rates in June and July, respectively. The specimen collection rate started rising sharply in August (11%; n = 22). In brief, most of the specimens (90%; n = 180) were collected during the study period between August and February. Further analysis of seasonality in terms of individual viral and bacterial pathogens will be elaborated in the later part of the article.

### Spectrum of bacterial pathogens in symptomatic cases

**Fig 2:**
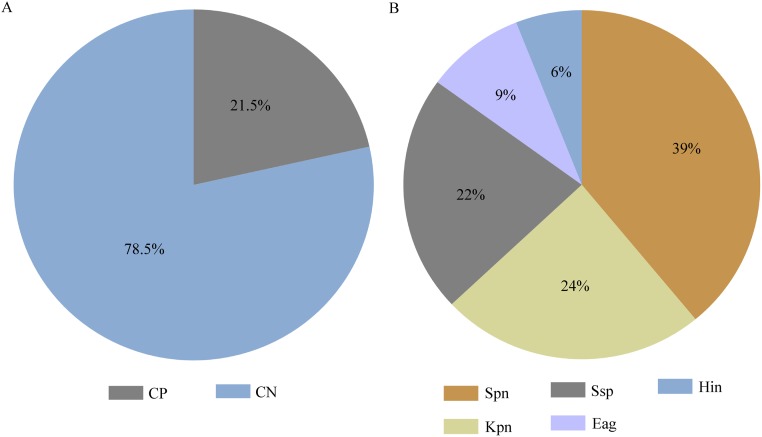
Detection and identification of bacterial pathogens isolated from 200 under-five children hospitalized with ARIs. Fig 2A shows the rates of culture positive and culture negative specimens. Fig 2B shows the proportion of different bacterial strains among culture positive specimens. In the Figure, CP = Culture Positive, CN = Culture Negative, Spn = *Streptococcus pneumoniae*, Kpn = *Klebsiella pneumaniae*, Hin = *Haemophilus influenzae*, Eag = *Enterobacter agglomerans*, Ssp = *Streptococcus spp*.

Next, we wanted to detect and identify different ARI-associated bacterial pathogens in under-five children. Conventional microbiological culture methods and biochemical tests were used to isolate and identify bacterial pathogens in the specimens. Among 200 symptomatic specimens, 43 specimens (21.5%) came out as culture positive (Fig 2A).

Taking into account of both single infection and coinfection cases of bacterial infections, 46 bacterial pathogens were isolated from symptomatic children; and the most commonly isolated bacteria were *S*. *pneumoniae* (18/46; 39%), followed by *K*. *pneumaniae* (11/46; 24%), *Streptococcus spp*. (10/46; 22%), *E*. *agglomerans* (4/46; 9%), and *H*. *influenzae* (3/46; 6%) (Fig 2B). On the other hand, 7% (2 out of 30) nasal swab specimens which had been collected from asymptomatic participants were culture positive and only *S*. *pneumoniae* was found in both cases. The data obtained from symptomatic cases demonstrate for the first time that *E*. *agglomerans* might play a role as one of the major bacterial pathogens (9%) of ARIs in under-five children in Bangladesh (Fig 2B).

### In vitro antibiotic sensitivity/resistance patterns for the isolated bacterial strains

Since antibiotic resistance, which is an interdisciplinary global concern, is emerging in an alarmingly increasing manner, the study also aimed to investigate antibiotic resistance patterns for the isolated bacteria. Table 1 shows that the *S*. *pneumoniae* isolates that had been found as the predominating bacterial agent colonizing the under-five ARI children were all sensitive to first line antibiotics including penicillin and ampicillin as well as levofloxacin, an aminoglycoside. In addition, 95% *S*. *pneumoniae* isolates showed sensitivity to ceftriaxone and 84% showed sensitivity to cefixime. However, a disappointing antibiotic resistance pattern for *S*. *pneumoniae* was observed for other antibiotics including erythromycin, azithromycin, and cotrimoxazole, showing resistance frequencies of 44%, 61%, and 89%, respectively. The antibiotic susceptibility test data demonstrate an alarming resistance level of *K*. *pneumaniae*, showing 100% resistance against azithromycin, 55% resistance against each of ceftriaxone, cefixime and ciprofloxacin; 27% and 45% resistance frequencies against Gentamicin and Tobramycin, respectively. Only carbapenem group of antibiotics including imipenem and meropenem had exhibited 100% sensitivity against *K*. *pneumaniae in vitro* (Table 1).

**Table 1:** Resistance pattern of the isolated bacteria to first line of antibiotics.

Antibiotics	Bacterial isolates				
	*Streptococcus pneumoniae* n = 18	*Klebsiella pneumaniae* n = 11	*Streptococcus spp*. n = 10	*Enterobacter agglomerans* n = 4	*Haemophilus influenza* n = 3
Penicillin	0	-	3 (30%)	-	-
Ampicillin	0	-	4 (40%)	-	1 (33%)
Erythromycin	8 (44%)	-	3 (30%)	-	-
Azithromycin	11 (61%)	11 (100%)	-	3 (75%)	0
Cotrimoxazole	16 (89%)	-	-	-	2 (67%)
Ceftriaxone	1 (5%)	6 (55%)	0	3 (75%)	1 (33%)
Cefixime	3 (16%)	6 (55%)	4 (40%)	3 (75%)	-
Levofloxacin	0	-	8 (80%)	-	-
Gentamicin	-	3 (27%)	-	2 (50%)	-
Tobramycin	-	5 (45%)	-	3 (75%)	-
Ciprofloxacin	-	5 (55%)	-	3 (75%)	-
Imipenem	-	0	-	1 (25%)	-
Meropenem	-	0	-	1 (25%)	-
Amoxyclave	-	-	-	-	1 (33%)
Chloramphenicol	-	-	-	-	1 (33%)

Modified disc diffusion method was used for profiling of antimicrobial susceptibility/resistance patterns. Bacterial strains were identified as either sensitive or resistant to an antibiotic based on the diameter of inhibition zone interpretative, as manifested by CLSI.

More concerning it is that one of the *K*. *pneumoniae* isolates showed resistance to all available antibiotics including all the first line and second line antibiotics (Table 2). Antibiotic resistance patterns for the next predominating ARI agent *Streptococcus* species (n = 10) were also alarming because there was 30% to 80% resistance frequency for all antibiotics except ceftriaxone. For *E*. *agglomerans*, 3 out of 4 isolates showed resistance to antibiotics like azithromycin, ceftriaxone, cefixime, tobramycin, and ciprofloxacin, whereas 2 isolates showed resistance to gentamicin. Even the carbapenems- imipenem and meropenem appeared ineffective against one *E*. *agglomerans* isolate, which showed resistance to all antibiotics. Finally, at least 1 of 3 isolates of *H*. *influenzae* showed some degrees of resistance to the antibiotics tested for antibiogram study.

**Table 2:** Resistance patterns of two multi-drug resistant bacteria to second line of antibiotics isolated in this study.

Bacteria	Name of the Antibiotics				
	Amikacin	Pipercilin/ Tazobactam	Carbenicillin	Polymyxin B	Netilmicin
*E*. *agglomerans*	Resistant	Resistant	Resistant	Resistant	Resistant
*K*. *pneumaniae*	Resistant	Resistant	Resistant	-	Resistant

Modified disc diffusion method was used for profiling of antimicrobial susceptibility/resistance. Bacterial strains were identified as either sensitive or resistant to an antibiotic based on the diameter of the inhibition zone interpretative, as manifested by CLSI.

Two multi-drug resistant bacterial strains—one *K*. *pneumaniae* isolate and one *E*. *agglomerans* isolate were resistant not only to all first line of antibiotics but also to all tested second line of antibiotics (Table 2).

In summary, if *in vitro* antibiotic resistance patterns found in this study match *in vivo* resistance patterns, it can be concluded that the antibiotic resistance levels have possibly crossed the danger line for *K*. *pneumoniae*, *Streptococcus* species, *E*. *agglomerans*, and *H*. *influenzae*, although *S*. *pneumoniae* still exhibits sensitivity to a good number of antibiotics. It should be mentioned here that two isolates of S. *pneumoniae* from asymptomatic ARI participants showed an antibiotic sensitivity pattern similar to that of *S*. *pneumoniae* isolates from symptomatic children (data not shown).

### Detection of respiratory viral pathogens in under-5 symptomatic ARI patients

Next, the study focused on the detection of viral pathogens (Tables 3 and 4). RT-qPCR method was adopted to detect a panel of 10 different respiratory viruses. About 79% (158/200) of enrolled ARI patients were laboratory-confirmed for viral infections with either single viral infections (54.5%; n = 109) or infections with more than one viruses (24.5%; n = 49). Although the number of viral infected patients was 158, the total number of virus detected in this study was 213 because of the presence of multiple viral pathogens in same specimen (Table 3). The most frequently detected virus was HRV (63/200; 31.5%), followed by RSV, HMPV, HBoV, HPIV-3 and adenovirus with detection rates of 31% (62/200), 13% (26/200), 11% (22/200), 10.5% (21/200), and 7% (14/200), respectively. Other respiratory viral pathogens that had been detected included Influenza A in 3 specimens, Influenza B in 1 specimen and HPIV-1 in 1 specimen. Among the bacterial culture positive patients described in Table 4, 17.5% (n = 35) had coinfections with viral pathogens.

**Table 3:** Prevalence and comparison of different respiratory pathogens between ≤ 1 year and 1–5 years age group.

Pathogens	Total Specimen (n = 200)	Age Group		P value	Risk Ratio (RR) [95% CI]	Odds Ratio (OR) [95% CI]
		≤ 1 year age (n = 157)	1–5 years age (n = 43)			
HRV	63 (31.5)	54 (34)	9 (21)	0.0922	1.167 [0.992–1.311]	1.981 [0.8854–4.43]
RSV	62 (31)	54 (34)	8 (19)	0.0473	1.167 [1.018–1.338]	2.294 [0.995–5.29]
HBoV	22 (11)	17 (11)	5 (12)	0.882	0.983 [0.774–1.245]	0.923 [0.319–2.663]
HMPV	26 (13)	20 (13)	6 (14)	0.834	0.977 [0.781–1.223]	0.9 [0.3372–2.403]
ADV	14 (7)	10 (6)	4 (9)	NA	-	-
HPIV-1	1 (0.5)	1 (1)	0	NA	-	-
HPIV-2	0	0	0	NA	-	-
HPIV-3	21 (10.5)	19 (12)	2 (5)	NA	-	-
Inf A	3 (1.5)	3 (2)	0	NA	-	-
Inf B	1 (0.5)	0	1 (2)	NA	-	-
Spn	18 (9)	14 (9)	4 (9)	NA	-	-
Kpn	11 (5.5)	8 (5)	3 (7)	NA	-	-
Ssp	10 (5)	7 (4.5)	3 (7)	NA	-	-
Eag	4 (2)	3 (2)	1 (2)	NA	-	-
HI	3 (1.5)	1 (1)	2 (5)	NA	-	-
Total specimens positive for pathogen	165 (82.5)	136 (87)	29 (67)	0.0034	1.374 [1.039–1.817]	3.123 [1.424–6.862]
Presence of multiple pathogens	72 (36)	62 (39.5)	10 (23)	0.049	1.698 [0.954–3.022]	2.154 [0.991–4.682]

NA = Not Applicable, Spn = *S*. *pneumoniae*, Kpn = *K*. *pneumaniae*, Ssp = *Streptococcus spp*. Eag = *E*. *agglomerans*, HI = *H*. *influenzae*. The percentage of the samples are in parentheses. P values are calculated with the 2-tailed χ^2^- test.

**Table 4:** The table displays the coinfection spectrum found in this study.

Coinfection with two pathogens	No. of Patients	Coinfection with three or more than three pathogens	No. of Patients
RSV+ *S*. *pneumoniae*	7	RSV+ HRV+ *K*. *pneumoniae*	2
RSV+ HRV	6	HRV+HPIV-3+ *K*. *pneumoniae*	2
HRV + HMPV	4	HRV+ *S*. *pneumoniae+ K*. *pneumoniae*	1
HRV + ADV	4	RSV+ *S*. *pneumoniae+ H*. *influenzae*	1
HRV + HPIV-3	5	RSV+ ADV+ *E*. *Agglomerans*	1
RSV+ *Streptococcus spp*.	3	RSV+ HPIV-3+ *S*. *pneumoniae*	2
RSV+ ADV	3	RSV+ HRV+ *S*. *pneumoniae*	1
HRV + HBoV	3	HRV+ HBoV+ *K*. *pneumoniae*	1
HRV + *K*. *pneumoniae*	2	RSV+ HBoV+ HPIV-1	1
HMPV+ *S*. *pneumoniae*	2	ADV+ HBoV+ HPIV-3	1
HRV+ *Streptococcus spp*.	2	HRV+ RSV+ *E*. *agglomerans*	1
HMPV+ *K*. *pneumoniae*	1	HRV+ HBoV+ *Streptococcus spp*.	1
HPIV-3+ ADV	1	HRV+ HPIV-3+ *S*. *pneumoniae*	1
RSV+ HBoV	1	HRV+ HBoV+ HPIV-3	1
HMPV + *Streptococcus spp*.	1	HPIV-3+ ADV+ RSV	1
*S*. *pneumoniae+ H*. *influenzae*	1	HRV+ RSV+ *K*. *pneumonia+ E*. *agglomerans*	1
HBoV+ HMPV	1	HRV+ HBoV+ HMPV+ HPIV-3	1
RSV+ *H*. *influenza*	1		
Influenza A+ HRV	1		
Influenza A+ ADV	1		
Influenza B + *S*. *pneumoniae*	1		
HMPV+ ADV	1		

### Age-dependent distribution of major viral and bacterial pathogens in under-5 ARI patients

For appropriate preventive measures and prompt interventions, determination of vulnerable age groups is important. Table 3 shows a comparison of distribution patterns of indicated respiratory viral and bacterial pathogens between <1 year and 1–5 year age groups.

As we can see here, the total number of specimens which had come out positive for pathogens was significantly higher in less than one year age group (87%) compared to 1–5 years age group (67%) (P = 0.0034, RR = 1.374 [1.039–1.817], OR = 3.123 [1.424–6.862]). The comparison of multiple pathogen detection rates between the specimens of these two groups also generated a significant P value (0.049) with RR and OR values of 1.698 [0.954–3.022] and 2.154 [0.991–4.682], respectively.

The majority of respiratory viral pathogens were highly prevalent in <1 year age group of children and the detections of HRV (54/63) and RSV (54/62) were almost confined to this age group. However, a significant P value was found in case of RSV (P = 0.0473, RR = 1.167 [1.018–1.338], OR = 2.294 [0.995–5.29]) only but not for HRV. Similar to HRV and RSV, the majority of HMPV (20/26), HBoV (17/22), adenovirus (10/14), HPIV-3 (19/21) and *S*. *pneumoniae* (14/18) and *K*. *pneumaniae* (8/11) were confined to less than 1 year old children, although the results did not show significant differences between the two age groups. Notably, pathogen detection rates dropped to almost 2% for all viruses for more than three-year old children (data not shown). Due to nil or very low levels of pathogen detection frequency (less than five), χ^2^- test was not performed for pathogens other than HRV, RSV, HBoV, and HMPV. Over all, the data suggest that under-one children are more likely to receive attention due to their vulnerability to infections by respiratory viral pathogens.

Coinfection is a frequently occurring phenomenon in numerous infections and this phenomenon has attracted a great deal of recent attention. Respiratory viral pathogens including influenza virus have been reported to predispose to secondary bacterial pulmonary infections [16], hence, data analysis targeting coinfection patterns was performed. Out of 46 bacterial isolates, 40 (87%) were associated with coinfections. On the other hand, 59% (126 out of 213) of total viral agents were accomplices in coinfections. In summary, the data indicate that coinfection is a major phenomenon in under-five children with ARI illness and the results are consistent with previously published reports on this subject.

For bacteria, the rates of coinfections of *S*. *pneumoniae*, *K*. *pneumoniae*, *Streptococcus* species, *E*. *agglomerans* and *H*. *influenza* were 94%, 90%, 70%, 75% and 100% respectively. On the other hand, the rates of coinfections of predominantly detected respiratory viral pathogens including HRV, RSV, HMPV, HPIV-3, HBoV, and adenovirus were 63.5%, 52%, 42%, 71%, 50% and 93%, respectively. In 72 coinfected cases, 52 showed a pair-wise fashion of co-occurrences of pathogens, whereas 18 showed pathogen co-occurrences in a tri-partner manner.

In two cases, up to four pathogens were detected in each specimen. Interestingly, coinfection partners of adenovirus were mostly confined to viruses including 5 RSV, 4 HRV, 3HPIV-3, 1 influenza A, 1 HMPV, 1 HBoV, whereas there was only one co-detection of bacterial isolates (*E*. *agglomerans*) with adenovirus (Table 4). Among 16 parainfluenza coinfection cases, 15 were HPIV-3 and 1 was HPIV-1. Although major HPIV-3 coinfection partners included HRV, RSV, adenovirus, *S*. *pneumoniae* and *K*. *pneumoniae*, one HPIV-1 case was co-detected with RSV and HBoV. On the other hand, 5 out of 11 HMPV coinfection partners included bacteria like *S*. *pneumoniae* (two), *K*. *pneumoniae* (one), and one *Streptococcus* species as well as viral pathogens including HRV (five), HPIV-3, HBoV and adenovirus.

Also, we analyzed coinfection data in terms of co-occurrences of 2 or more pathogens in the host, such as viral-viral, viral-bacterial, viral-viral-bacterial, and viral-bacterial-bacterial patterns. As we can see in Table 4, HRV was observed as the most predominant coinfection partner, showing its presence with almost all of the pathogens. The major coinfection viral partners of HRV included RSV (six), HPIV-3 (five) HMPV (four), adenovirus (four), HBoV (three), and Inf A (one) viruses. In addition, HRV was co-detected in 9 out of 10 *K*. *pneumoniae* infection cases, whereas it was co-detected in only five *S*. *pneumoniae* cases. After HRV, RSV was the second most predominant pathogen and it was co-detected mainly with *S*. *pneumoniae*, *K*. *pneumoniae*, *Streptococcus* species, *E*. *agglomerans*, and *H*. *influenzae* and a total of 20 bacterial isolates had been found with RSV.

Other than co-detection with bacteria, RSV was also co-detected with adenovirus in 5 specimens, HPIV-1 and HPIV-3 in 1 and 3 cases, respectively. HMPV was more likely to be identified in single infections, whereas adenovirus, *S*. *pneumoniae*, and *K*. *pneumaniae* were mostly identified in coinfection cases. Although HBoV was equally detected in both single and coinfection cases, 63.09% of HBoV which had been found in coinfected cases were observed as a partner with HRV (7 cases). Among 3 Influenza A positive cases, one had been detected without any coinfection partner and 2 had coinfections with adenovirus and HRV (shown in Table 4). The only case of Influenza B virus had coinfection with *S*. *pneumoniae*.

**Fig 3:**
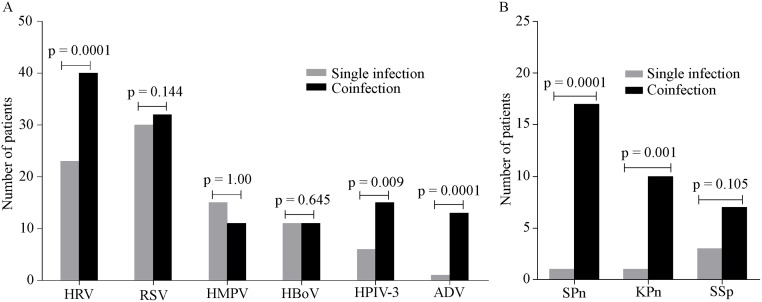
Comparison of frequencies of each pathogen identified in single infections and coinfections. Fig 3A compares the frequencies of viruses and Fig 3B compares the bacterial pathogen frequencies in single infections and coinfections. Fisher's exact test was performed for each group. P< 0.05 was considered significant. SPn = *S*. *pneumoniae*, KPn = *K*. *pneumoniae*, SSp = *Streptococcus* species.

Next, we wanted to analyze the distribution patterns of major viral and bacterial pathogens between single infections and mixed infections. Fig 3A indicates that the numbers of coinfected cases of HRV, HPIV-3, and adenovirus were significantly higher than that of single infection cases, generating significant P values of 0.0001, 0.009, and 0.0001, respectively. In cases of RSV, HMPV, and HBoV, no significant differences in frequency were observed between cases of single infections and coinfections for each viral agent. We also analyzed coinfection status in terms of bacterial pathogens. As we can see in Fig 3B, the numbers of cases where *S*. *pneumoniae* and *K*. *pneumoniae* had been found as coinfection partners were 17 (n = 18) and 10 (n = 11), respectively.

The comparisons between frequencies of single infections and coinfections for *S*. *pneumoniae* and *K*. *pneumoniae* generated significant P-values of 0.0001 and 0.001, respectively. For *Streptococcus* species, although coinfection cases were higher than single infection cases, the difference did not generate a significant P value. Higher rates of bacterial coinfections by *S*. *pneumoniae* and *K pneumonia*e in synergy with viruses may indicate that predominant ARI viruses predispose their hosts to secondary infections by bacteria.

### Detection of respiratory pathogens in asymptomatic cases

Recent studies of asymptomatic ARI cases reported the frequent presence of respiratory pathogens like HRV, adenovirus, *S*. *pneumoniae* etc. In this study, we collected 30 asymptomatic specimens. 43% (n = 13) of those asymptomatic children were positive for pathogen(s). However, the detection rate of pathogens in symptomatic specimens (82.5%) was significantly higher than that of in asymptomatic specimens (P< 0.0001) (Table 5).

**Table 5:** Comparison of pathogen detection rates between asymptomatic and symptomatic cases of under five children.

Pathogens	Asymptomatic Specimens n = 30	Symptomatic Specimens n = 200	P value
HRV	7 (23)	63 (31.5)	0.404
RSV	2 (7)	62 (31)	0.004
HMPV	1 (3)	26 (13)	0.219
ADV	3 (10)	14 (7)	0.471
Spn	2 (7)	18 (9)	1.00
Specimens positive for pathogen	13 (43)	165 (82.5)	<0.0001
Presence of multiple pathogens	2 (7)	72 (36)	0.0007

The table shows the comparison of detection rates of only those pathogens which had been detected in both asymptomatic and symptomatic cases. Values in parentheses indicate percentages. P values were calculated with 2-tailed Fisher's exact test. Spn = *S*. *pneumoniae*.

HRV was the most prevalent pathogen in both symptomatic (31.5%) and asymptomatic (23%) cases. However, the difference in detection rates of HRV between symptomatic and asymptomatic cases did not generate a significant P-value (P = 0.404). For adenovirus, the rate of pathogen detection was higher in asymptomatic specimens (10%) than that of in symptomatic specimens (7%), although the difference in detection rate between these two categories of specimens was not statistically significant. Similarly, for HMPV and *S*. *pneumoniae*, the difference in detection rate between the symptomatic and asymptomatic attacks were not significant (P = 0.219 and P = 1.00, respectively). On the other hand, RSV detection rate was significantly higher in symptomatic specimens than asymptomatic specimen (P = 0.004), suggesting the potential involvement of RSV in ARI illness. Finally, multiple pathogens were detected in only 7% (2/30) of asymptomatic specimens and this detection rate was significantly lower than the detection rate of multiple pathogens in symptomatic specimens (36%, P = 0.0007), implying possible pathogenic role of multiple pathogens in ARI symptoms. It is mentionable here that we couldn’t detect any other bacteria other than two isolates of *S*. *pneumoniae* in 30 asymptomatic cases.

### Seasonality of viral and bacterial pathogens in terms of hospitalization of ARI patients

**Fig 4:**
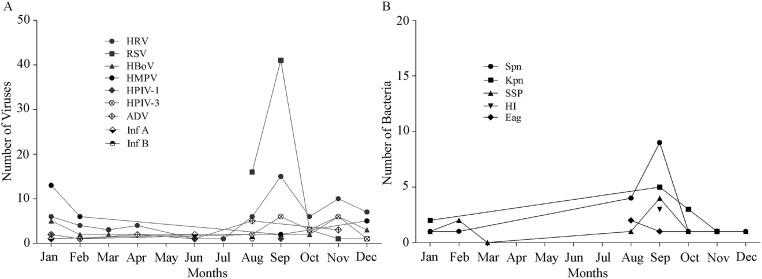
Monthly distribution of respiratory pathogens detected in this study. Fig 4A shows the distribution of the detected viruses and Fig 4B displays the distribution of the isolated bacteria, in nasal swab specimens collected in different months during the study period. In 4A, Inf A = influenza A, Inf B = influenza B and in 4B, Spn = *S*. *pneumoniae*, Kpn = *K*. *pneumaniae*, HI = *H*. *influenzae*, Eag = *E*. *agglomerans*, Ssp = *Streptococcus spp*.

In Fig 1, we observed seasonal variation in terms of specimen collection rates but not the actual pathogen detection rates. Therefore, it is important to know true seasonality for each of the individual viral and bacterial pathogens. As we can see in Fig 4A, HRV reached its highest peak (n = 15) in September and 2^nd^ highest peak in November (n = 10). In addition, there were six HRV positive specimens in October. From January to August, HRV circulation was roughly constant, indicating HRV transmission throughout the year. Similar to HRV, the number of RSV-infected patients reached its peak in September, when 41 (66%) of 62 patients were hospitalized. On the other hand, the hospitalization rates of RSV patients were 26% (n = 16), 4.8% (n = 3), 1.6% (n = 1), and 1.6% (n = 1) in August, October, November, and December, respectively, whereas there were no detections of RSV-infected patients in other months. These findings suggest that RSV circulation was highly confined in August and September. In contrast to RSV, HMPV circulation reached its peak (50%, n = 13) in January, followed by 23% (n = 6), 19% (n = 5), and 8% circulation in February, December, and September. There were no enrollments of HMPV-infected patients in other months. After HRV, RSV, and HMPV, HBoV had the fourth highest detection rates in terms of numbers of HBoV-infected patients and all of them were detected between September and March.

HPIV-3 showed 2 peaks-1 in September and another in November, each representing 6 patients, indicating hospitalization of 56% (n = 12) of HPIV-3-infected patients in these 2 months. The rest 9 of 21 HPIV-3 patients were hospitalized in August, October, December, January, and February with hospitalization rates of 10% (n = 2), 14% (n = 3), 5% (n = 1), 10% (n = 2), and 5% (n = 1), respectively. On the other hand, hospitalization rates of adenovirus-infected patients were 36% (n = 5), 21% (n = 3), 10% (n = 2), 5% (n = 1), 10% (n = 2), and 5% (n = 1) in August, November, January, February, April, and June, respectively. Other viruses of the viral panel including HPIV-1, HPIV-2, influenza A, and influenza B appeared in small numbers of patients and were not informative for determination of seasonality. Next, we analyzed seasonality of ARI-causing bacterial pathogens. Fig 4B demonstrates that the most prevalent bacterial pathogen *S*. *pneumoniae* started appearing in August with four patients and the number of infected patients reached its peak in September, showing hospitalization of 9 (50%) out of 18 patients. There was only one patient enrolled in each of the months of October, November, December, January, and February for *S*. *pneumoniae*-associated hospitalization. On the other hand, the number of second most prevalent bacterial pathogen *K*. *pneumoniae* showed its peak circulation in September, as manifested by 5 cases (46%) of *K*. *pneumoniae*-associated hospitalization in this month, followed by 3 (27%), 1 (9%), and 2 (18%) hospitalized cases in October, November, and January. Like *S*. *pneumoniae* and *K*. *pneumoniae*, *Streptococcus* species had a peak circulation in September with 4 (40%) hospitalized patients.

The rest 6 of the 10 patients infected with *Streptococcus* species were hospitalized in August (n = 1), October (n = 1), November (n = 1), January (n = 1), and February (n = 2). Interestingly, circulation of all 3 (100%) *H*. *influenzae* isolates were confined in September. Unlike the above-mentioned bacterial pathogens which had a peak circulation in September, *E*. *agglomerans* had a peak circulation in August with only 2 (50%) patients.

Thus, the data suggest that all of the bacterial isolates except *E*. *agglomerans* had a peak circulation in September, which is also the peak season for the most predominant viral agent RSV and this phenomenon of simultaneous occurrences of viral and bacterial pathogens may facilitate huge numbers of bacterial coinfections with RSV.

## Discussion

The study demonstrates for the first time the simultaneous detection of viral and bacterial etiologies of acute respiratory infections (ARIs) in under-5 children in hospital-care settings in Dhaka, Bangladesh. Major focal points of the study included (a) determination of seasonal variation in terms of ARI specimen collection rates as well as seasonality of isolated ARI pathogens, (b) in vitro antibiotic resistance/sensitivity assay for bacterial pathogens, (c) coinfection patterns, (d) age-dependent distribution patterns of pathogens, and (e) a comparison of the detection rates of respiratory pathogens between symptomatic and asymptomatic cases. Knowing the seasonal variability of pathogens would help the parents or guardians of the children as well as physicians to adopt some preventive measures. Antibiogram study was designed to know the condition of antibiotic resistance/sensitivity patterns of major ARI bacterial pathogens to avoid unnecessary use of antibiotics to treat ARI patients, which also might help control of the rapid emergence of antibiotic resistant strains. Analysis of coinfection status was performed to identify major coinfection partners in terms of virus-bacteria, virus-virus, bacteria-bacteria, virus-bacteria-bacteria, and virus-virus-bacteria. Especially specific identification of bacteria in a coinfection case may help the physicians to choose appropriate antibiotics and additional medications because bacterial coinfection, especially superinfection has been reported to associate with huge fatal outcomes and deaths of under-five ARI patients. On the other hand, age-dependent distribution patterns of pathogens are useful to identify vulnerable age groups.

Information on seasonal variability would be helpful to create an extra awareness among the community and health professionals including physicians and nurses. Preventive measures including personal and in-house hygiene and cleanliness, and proper ventilation may be adopted across the peak ARI season. Other preventive measures include avoiding crowded public places and making a habit of frequent hand washing etc. Our data demonstrate that the majority of 200 symptomatic specimens were collected between August and February corresponding to post-monsoon and winter. In this region, the temperature mostly remains low in these months and it is commonly known that respiratory illness incidence rate is higher in winter. Winter season has bad reputation not only for upper respiratory tract infections caused by a large number of viruses but also for lower respiratory tract infections including pneumonia [17, 18]. Our study demonstrates that the most predominantly detected viral pathogen HRV was circulated throughout the year without showing any clear seasonal distribution. On the other hand, the peak circulation of the second most prevalent viral pathogen RSV was seen in September. HMPV showed its seasonal peak in January. The findings regarding winter seasonality of viral pathogens are thus consistent with previous studies of respiratory viral pathogen detections in Bangladesh [10, 19]. Similar to the majority of viral pathogens, the bacterial pathogens including *S*. *pneumoniae* and *K*. *pneumoniae*, showed their peak circulations in September. So, the vulnerable group of children of the country requires appropriate preventive measures against ARI across the post monsoon and winter. However, this was a one-year study and the time period is meager to substantiate any seasonality of the pathogens.

Irrespective of single or coinfections, our experimental approach could successfully identify viral and/or bacterial pathogens in 165 (82.5%) symptomatic cases of participants, whereas 35 cases remained undetected of any pathogens. A community-based study conducted in Bangladesh could identify respiratory viral pathogens in only 66% of ARI cases and the detection rate was lower than the detection rate calculated for this study [10]. However, the study did not consider bacterial pathogens. On the other hand, the studies of respiratory infections among children of Zambia and Madagascar reported the detection rates of respiratory pathogens in 76.8% and 74.6% specimens, respectively, and these studies focused on the detection of both viral and atypical bacterial pathogens [20, 21]. A similar study conducted in China demonstrated 67.5% overall pathogen detection rate [22]. These all suggest that our findings in terms of pathogen detection are consistent with the reported data of similar studies.

Although the viruses are the most common etiological agents of ARIs, a wide variety of bacterial pathogens are also associated with ARIs either as single or mixed agents with viruses. The bacterial etiology is not investigated in most of the ARI-associated studies. Even in countries like the USA where pneumonia surveillance is routinely conducted, the bacterial etiology is not recorded in 65–85% of hospitalized pneumonia cases [23, 24]. It cannot be rule out that the presence of bacteria as coinfection partners with viruses may complicate the clinical symptoms of ARI illness leading to fatal outcomes. As such, the study was designed to focus not only on bacterial detection but also on antibiotic sensitivity/resistance patterns of the isolated bacterial strains.

Our microbiological culture method could detect bacterial pathogens in 21.5% cases, although the majority of bacterial pathogens had been co-detected with viruses. The most commonly isolated bacteria were *S*. *pneumoniae* (39%) and *K*. *pneumaniae* (24%). *H*. *influenzae* was identified in 6% cases. Along with *S*. *pneumoniae* and *K*. *pneumaniae*, we could also detect *Streptococcus* spp. (22%) and *E*. *agglomerans* (9%). *S*. *pneumoniae* is a predominant cause of <5 child mortality and despite the vaccination program, it is responsible for at least 18% of severe respiratory episodes and 33% of deaths worldwide [25]. Other studies conducted in Kenya, Zambia, Nepal, and Brazil reported *S*. *pneumoniae* as the most frequently isolated bacteria and the isolation rate ranged from 15.8–54.8% [26–29]. *K*. *pneumoniae* had been described as the most common cause of lower respiratory tract infection in Jordan and India [30, 31]. It is known that bacterial colonization is mostly facilitated by physical damages of respiratory cells caused by viral infection. However, Yu et al. reported that bacterial colonization following viral infection has no effect on clinical manifestation of ARIs [32]. Furthermore, the role of bacterial pathogens in ARI symptoms is ambiguous as recent studies of asymptomatic ARI cases reported frequent bacterial colonization [33] and our study also found comparable detection rates of *S*. *pneumoniae* in both asymptomatic and symptomatic children. Many of the bacteria found as coinfection partners with virus in this study, are pathogenic. When bacteria enter a body, the host must elicit immune responses against the invading organisms. However, the immune responses are not always beneficial and sometimes these responses can be harmful, such as in case of hyper immune responses. Even the immune responses elicited by one organism may compromise the immune responses elicited by another organism. Although the pathogenic role of bacterial coinfections with viruses is unclear, they might have involvement in disease pathogenicity. One study reported that, colonization of pathogenic bacteria in airway stimulate topical immune responses [34] and this phenomenon may play important role in compromising host immune responses. Most of the bacteria isolated during the study period including *S*. *pneumoniae*, *K*. *pneumaniae*, *H*. *influenzae*, and *E*. *agglomerans* fall in either community-acquired or nosocomical categories, suggesting appropriate preventive measures could limit bacterial involvement in ARIs.

Increasing antibiotic resistance to commonly prescribed antibiotics makes bacterial infections a major threat to public health worldwide. As we have described in result section, our *in vitro* antibiotic resistance patterns were concerning in the sense that most of the isolates except *S*. *pneumaniae*, such as *K*. *pneumaniae*, *Streptococcus* species, *H*. *influenzae*, and *E*. *agglomerans* exhibited alarming levels of resistance against the majority of available first line of antibiotics. Even two of 15 multidrug-resistant isolates (*K*. *pneumaniae* and *E*. *agglomerans*) displayed resistance not only against all the first-line of antibiotics but also against all the second-line of antibiotics. Despite the advances in therapeutic and preventive measures, the emerging resistance to the antibiotics is a growing concern among clinicians and other health professionals worldwide. The indiscriminate and unreasonable use of antibiotics have contributed to the emergence of resistance, which may turn out to be a leading cause of morbidity and mortality in the developing countries. A tailored antibiotic treatment is in demand to tackle this major issue, but the dearth of information regarding the etiological agents and antibiotic sensitivity patterns in countries like Bangladesh have made it difficult. The information regarding antibiotic resistance patterns provided here in this study is expected to raise alertness among physicians, community people, as well as among policy makers of public and private sectors in the country.

Single-plex reverse transcription real-time PCR (RT-qPCR) method could detect the presence 213 viruses in 158 (79%) children. The findings corroborate the concept of viruses being the most common causative agent of ARIs and the data are consistent with hospital-based findings in Bangladesh reported by Homaira *et al*. [11] and the comparable `s were also published from Laos and Egypt [35, 36]. However, many studies in other regions including Brazil, Niger, Zambia, Spain, and Mexico reported higher viral detection rates ranging from 65–87% [26, 37–40]. The disparity in viral detection rates among these study findings might be due to variation in numbers of viruses that were included in the viral panel of each study group. Our viral panel included ten common respiratory viruses including respiratory syncytial virus (RSV), human rhinovirus (HRV), influenza A and B viruses, human parainfluenza viruses type 1 to 3 (HPIV1, HPIV2, HPIV3), human metapneumo virus (HMPV), human bocavirus (HBoV), and adenovirus. However, the panel did not include coronavirus which may account for a certain percentage of infections. 31.5% specimens were positive for HRV, which was predominantly detected as a viral pathogen. However, the incidence rate of HRV was also higher in asymptomatic cases (23%). Interestingly, the detection rate of adenovirus was higher in asymptomatic cases (10%) than the symptomatic specimens (7%). A study conducted by Singleton et al. also frequently identified HRV and adenoviruses in healthy children [41], which makes it difficult to make any inference regarding the association of these two viruses with respiratory illness. RSV was the second most prevalent virus identified in 31% cases. However, unlike HRV, RSV has been reported as a recurrent cause of ARI in both children younger than five years old and adult, with incidence reaching 26% (Laos PDR), 33% (Mexico), 35% (Niger), 37% (Brazil), 51% (Germany), 57% (India), and 76% (Spain) of the investigated cases [35, 37–40, 42, 43]. RSV can cause both upper and lower respiratory tract infections. After RSV, HMPV (13%), HBoV (11%) and HPIV-3 (10.5%) came out as the third, fourth, and fifth most prevalent viral agents. However, we observed relatively low pathogen detection rates for HPIV-1, HPIV-2, influenza A, and influenza B viruses. Thus after RSV, HMPV, HBoV, and HPIVs came out as the frequent causes of hospitalization of children with respiratory illness and the findings are consistent with other published data [24]. Approximately 70–89% cases of bronchiolitis had been reported to associate with RSV infection and 3–25% with parainfluenza viruses and there werek very few cases of bronchiolitis which could be attributed to human influenza virus and adenovirus [44, 45]. Several studies have described frequencies of 3.3–19% for HMPV infections in hospitalized children with respiratory tract infections [7, 44–47], although HMPV has been known to cause severe respiratory infections in the elderly [48, 49]. The frequency of HBoV identified in this study (11%) was also comparable with HBoV detection rates in Spain (9.9%) and Vietnam (7.2%) [50, 51].

Age-dependent distribution of respiratory virus data of our study shows that viral infection is mostly concentrated in less than one-year age group particularly RSV as a substantial threat. The frequency of pathogen positive specimens and incidence of coinfections were significantly higher in under-one children, which makes it the most vulnerable age group for ARIs. Additionally, around 87% (54/62) of the RSV cases were detected in children less than one year of age. The frequency of RSV infections in this age group seems to be a consequence of multifactorial complex events, involving especially immunological factors. Maternal antibodies are a vital means of protection to infants against respiratory viruses as they have a relatively immature immune system to mount an effective immune response. RSV antibodies are found in all adults and children older than three years of age. At one year of age, 25–50% of children have antibodies against RSV, indicating the high frequency of this infection at a young age [52] and then the incidence of RSV infections decreases with age, probably because of the development of anti-RSV immunity which is boosted during each subsequent re-infection [53]. Currently, there are no effective antivirals or vaccines against RSV infections. Several RSV vaccines are being evaluated in clinical trials and have yet to be licensed [54].

The study identified 35 cases with viral-bacterial mixed infections where RSV and *S*. *pneumoniae* were the predominant viral and bacterial partners, respectively, followed by HRV and *K*. *pneumoniae* duo. Each of HRV, RSV, *S*. *pneumoniae*, and *K*. *pneumoniae* showed a seasonal peak in September and both *S*. *pneumoniae*, and *K*. *pneumoniae* had significant participation in mixed infections demonstrating that viral infection renders bacterial colonization. Higher rates of bacterial infections by *S*. *pneumoniae* and *K*. *pneumoniae* in synergy with RSV, HRV, and other viruses may indicate that predominant ARI viruses predispose their hosts to secondary infections by bacteria. The other major viral partners of bacterial mixed infections included HMPV and HPIV. The mechanisms by which viruses influence bacterial colonization and invasion are very diverse. It has been shown that viral infections may predispose to bacterial super-infections by favoring bacterial attachment sites on nasopharyngeal epithelial cells and through increased mucous production that promotes bacterial growth [55, 56]. The real clinical significance of these infections has not yet been fully elucidated. Under the circumstances, emphasis should be given on deciphering the role of bacterial mixed infections in promoting disease pathology. The presence of pathogens in asymptomatic children made the aspect more puzzling. However, a case control study hypothesized that there could have a subclinical infection in asymptomatic cases which may act as a pathogen transmission source [57]. It is also important to remember that the detection rate of mixed infection is dependent mostly on methodology and number of pathogens that are being screened. Despite differences in methodologies, the similarity in rates of hospitalized viral respiratory illness in different countries suggests that respiratory viruses indeed are important contributors of childhood hospitalization around the world.

There are several shortfalls of the study including (a) shorter study period, (b) low number of both symptomatic and asymptomatic specimens, (c) exclusion of coronavirus from viral panel selected for RT-qPCR assay, (d) lack of some clinical information and follow-up study, (e) use of nasal swabs instead of nasopharyngeal swabs for specimen collection, (f) exclusion of ARI-causing fungal pathogen detection. Thirty-five out of 200 specimens remained undetected of any viral or bacterial pathogens. It is highly likely that the inclusion of fungal pathogen detection system and the addition of coronavirus in the viral panel would increase the rate of pathogen detection to some extent. In addition, the detection rate of bacterial pathogens could have been higher if PCR or qPCR had been used instead of culture method as the former one has higher sensitivity. However, we wanted to isolate all types of possible organisms and the use of specific primers would have confined the range of organisms. For example, *E*. *agglomerans* which has been rarely reported as a causative agent of ARIs was isolated in 4 (2%) nasal swab specimens in this study. The nasopharyngeal aspirate (NPA) has been considered to be the best sampling technique, but the procedure of obtaining an NPA specimen is uncomfortable and often frightening for young children. It is also unpleasant for the medical staff, who have to carry out the procedure with a struggling, crying, and coughing child. However, pathogen detection sensitivity did not differ significantly between nasal swab and NPA approaches of specimen collection when sensitive real-time PCR method was used to detect pathogens [58, 59]. In the study, only those viruses that are frequently detected in Bangladesh were listed in the viral panel. Even with these limitations, the study provides important preliminary data that can be used for more focused surveys in a larger population. The frequency of viral infection should be taken into account by pediatricians to avoid the irrational use of antibiotics.

In summary, ARI incidence, as well as pathogen detection rates, were higher during post-monsoon and winter in Bangladesh. HRV and RSV were the predominant viral causative agents in under-five ARIs. On the other hand, *S*. *pneumoniae* came out as the most prevalent bacterial causative agent. Children aged less than one year of age were most susceptible to infections including coinfections.
